# e-Cigarette Cessation: Content Analysis of a Quit Vaping Community on Reddit

**DOI:** 10.2196/28303

**Published:** 2021-10-25

**Authors:** Laura Struik, Youjin Yang

**Affiliations:** 1 School of Nursing Department of Health and Social Development University of British Columbia Okanagan Kelowna, BC Canada

**Keywords:** qualitative research, electronic nicotine delivery systems, vaping, cessation, social media

## Abstract

**Background:**

e-Cigarette use, also known as vaping, has increased dramatically over the past few years, especially among younger demographics. However, researchers have found that a large number of e-cigarette users want to quit. Little is known about the unique aspects of vaping cessation, which is critical to informing the development of relevant resources and interventions for e-cigarette users who want to quit. Social media forums such as Reddit provide opportunities to understand the experiences of behavior change such as quitting vaping from the perspective of end users.

**Objective:**

This study aims to examine a quit vaping subreddit to understand how e-cigarette users are experiencing and approaching vaping cessation. Specifically, we examine methods used to approach quitting, reasons for quitting, and barriers and facilitators to quitting.

**Methods:**

A total of 1228 posts were collected. The posts were inductively coded to generate categories and subcategories using conventional content analysis. Data were analyzed using the NVivo 12 qualitative data analysis software.

**Results:**

Most users reported a preference for approaching quitting through gradual reduction, particularly through the use of their own devices by tapering the nicotine content. Their reasons for quitting were primarily related to experiencing negative physical consequences associated with vaping, especially in relation to their lungs (eg, tight chest), and tired of feeling stuck to the vape because of nicotine addiction. Top barriers to quitting were related to withdrawal symptoms and intensity of addiction. The top facilitators to quitting were related to using distraction techniques (eg, hobby, gaming, and mindfulness exercises), as well as having a positive mindset.

**Conclusions:**

The findings of this study reveal unique aspects that encompass the process of quitting vaping. These findings have significant implications for both policy and intervention development.

## Introduction

Vaping with nicotine, or using e-cigarettes, has become a popular global phenomenon in recent years. Compared with 2011, whereby 7 million people worldwide used e-cigarettes, the number of e-cigarette users had increased to 41 million in 2018 [1], with youth and young adults representing the largest users of e-cigarettes [2]. Despite how vaping has grown in popularity, recent evidence indicates that a large number of e-cigarette users actually want to quit [3-6]. A focus group study in Canada found that >40% of youth and young adults who vaped indicated a strong desire to quit, and >40% had tried to quit in the past year [5]. Another study in the United States found that 45% of youth were interested in quitting, and 25% had tried to quit in the past year [4].

Although it is encouraging that many e-cigarette users want to quit, there remains a lack of cessation services to support their quit efforts. Researchers acknowledge that most cessation advice mimics smoking cessation [7] and that there is a dearth of resources tailored specifically to vaping [4,5]. The 2020 Surgeon General’s report on smoking cessation called for research to develop and understand safe and effective e-cigarette cessation guidelines and interventions [8]. A critical step toward developing effective cessation resources is to first understand the experiences and preferences of e-cigarette users as it relates to quitting vaping. However, no studies to date have examined how vapers quit and what might help them with quitting.

Web-based forums serve as particularly fruitful platforms for gathering information on end user experiences and preferences. Reddit, the most comprehensive forum on the internet [9], has several e-cigarette–related discussion topics. The use of social media such as Reddit to understand vaping cessation experiences is particularly important given that engagement on social media platforms for sharing and gathering health information is highest among youth and young adults [10,11]. Researchers have studied Reddit discussions to generate important information about e-cigarette flavors [12], the health effects of e-cigarette use [13,14], e-cigarette use patterns [9,15,16], reasons and attitudes surrounding underage e-cigarette use [17], and underage buying and selling [18]. However, no researchers have examined the quit vaping communities that are now on Reddit. This user-generated content around vaping holds significant potential to inform best practices for cessation guidelines and services. Therefore, we examined a quit vaping subreddit to understand how e-cigarette users experience and approach vaping cessation. Specifically, we examined methods for approaching quitting, reasons for quitting, and barriers and facilitators to quitting.

## Methods

### Overview

Similar to other studies involving Reddit [17-19], the examined subreddit contains publicly available threads and comments. No personal information (eg, account details) was included, and the usernames of the Reddit posters were not presented in this study. As such, this study was classified by the University of British Columbia Okanagan’s Behavioural Research Ethics Board as research not involving human subjects and was, therefore, not subject to institutional review board jurisdiction.

### Data Collection

We examined the subreddit r/QuitVaping community for this study. This subreddit was created in 2015 and designed to help vapers motivate each other to quit. This subreddit contained 4700 subscribers. Posts (including original posts and all responses) were collected retrospectively during a 4-week period (August 1, 2020, to August 31, 2020) by using the *top posts of this month* feature. The *top posts of this month* means that the posts with the most upvotes over this period were selected for analysis. This approach to data collection is supported by other content analyses of subreddit communities (eg, see work by Sowles et al [19]). The posts were first copied and pasted onto Microsoft Word, which were then uploaded onto the NVivo 12 qualitative analytic software (QSR International Pty Ltd) for coding. A total of 1228 posts were collected and analyzed.

### Data Analysis

The posts were inductively coded to generate categories and subcategories using conventional content analysis [20]. Both authors engaged in collaborative coding via UBC Zoom to code three original posts and 10 responses (13 total), which resulted in the assignment of 44 codes. The authors developed a coding legend and assigned preliminary codes directly onto the Microsoft Word document via the *comments* feature. The authors then independently reviewed an additional set of three original posts and eight responses (11 total). Each author assigned >35 codes. Only six discrepancies in coding were noted, which were then discussed, resolved, and worked into the coding framework. The second author then independently coded the remaining posts, consulting with the first author regularly as needed. Once all the posts were coded, authors met again and refined and collapsed the framework with subcategories under the four major categories of quitting methods, reasons for quitting, barriers to quitting, and facilitators to quitting.

## Results

### Users

In total, 318 unique users were represented in this sample of posts. A little less than 10% (29/318, 9.1%) of the represented sample indicated their age, with most (24/29, 83%) of them in the young adult age range (aged between 18 and 35 years), and the remaining outliers were aged <18 years (2/29, 7%) and >35 years (3/29, 10%). This may indicate a relatively young sample overall.

### Posts

Of the 1228 posts, 189 (15.39%) were original posts, and 1039 (84.61%) were responses to these posts. The average number of responses per original post was 5.5 (SD 5.55), and the range in the number of comments per post was 0-50, with most posts within the range of 5-10 and only one post with 50 comments. This one post is noteworthy, as the engagement was garnered through a call to action for mutual support and accountability. The post is as follows:

Everyone who is actively quitting/trying to quit/already did quit and wants to keep it that way, comment on this post. I will send each of you a message on reddit every single day checking up on you, and sending you motivational things to aid in your journey. Only asking that you try and do the same for me :) let me know!!!

The data within these 1228 posts are broken down into the following four categories: quitting methods, reasons for quitting, barriers to quitting, and facilitators to quitting (Table 1). The most represented categories of post content in this data set were related to barriers and facilitators to quitting.

**Table 1:** Representation of post categories (N=1228).

Category	Values, n (%)
Quitting methods	175 (14.25)
Reasons for quitting	211 (17.18)
Barriers to quitting	441 (35.91)
Facilitators to quitting	570 (46.42)

#### Quitting Methods

e-Cigarette users’ method of quitting was reflected in 14.25% (175/1228) of posts. Most of these posts (117/175, 66.9%) reflected a preference for a gradual reduction approach. This was accomplished primarily by tapering the nicotine concentration (55/175, 31.4%), which was followed by the use of different sources of nicotine replacement therapy (NRT; 33/175, 18.9%; eg, patches, gum, lozenges, toothpicks, and Nicorette inhalers). The most popular NRT used were patches and gum, which are often used together. Overall, 13 users reported using nicotine pouches to help them quit, and 6 stated using the vape less frequently. The *cold turkey* approach was represented in 33.1% (58/175) of the posts.

#### Reasons for Quitting

The reasons for quitting vaping were provided in 17.18% (211/1228) of posts (Table 2). There were eight reasons for quitting, as listed by the posters. The number one reason for quitting vaping was experiencing negative physical health effects of vaping, which included shortness of breath, chest pain, wheezing, coughing, sore throat, poor oral health, poor skin health, and poor sleep quality. The second most commonly cited reason to quit was that individuals were tired of feeling stuck to their e-cigarettes, indicating that they wanted to be free to live their lives without a dependency on a vaping device. The other most common reasons for quitting are related to the mental health and financial cost of vaping. These top reasons were then followed by a desire to improve physical health (eg, to improve running time), accommodate life changes (eg, having a baby), to gain time they would be otherwise using to vape or think about vaping, and to improve their intimate relationships (eg, spending time with partners who do not vape or smoke).

**Table 2:** Reasons for quitting (N=211).

Reason	Values, n (%)	Representative quotes
Experiencing negative physical health consequences	59 (28.0)	“I vaped for 3 years too, very consistently, and I really didn’t have a problem with it until I felt the health issues—constant dry throat, never ending phlegm, tonsil stones, fatigue, swollen lips (from vape juice spitting), irritability, and horrible acne. I also felt like I hadn’t been myself for awhile.”
Tired of feeling stuck to the vape or nicotine	47 (22.3)	“Last night, I had a moment of clarity. My whole life basically revolves around my vape. It's my first thought when I wake up every morning, I have to constantly have it with me, and everything ends with a vape. Anytime I’m without it, I’m just looking forward to the time I can use it. It’s honestly hard to imagine life without it, but I know I don’t want to be a 70-year-old vaper.”
Experiencing negative mental health consequences	28 (13.3)	“I’m fed up with feeling that anxiety and irritability when I’m not vaping.”
Financial loss	28 (13.3)	“I had to get to a point where I hated what it was doing to me and my finances.”
Wanting to improve physical health	17 (8.1)	“I was hoping quitting would help me with my run times before being winded (which currently doesn’t take too long).”
Need to adapt to life changes	17 (8.1)	“But with Covid and everything happening, I want to kick this addiction to the curb.”
Improve use of time	6 (2.8)	“Vaping sucks so much it makes you procrastinate, it makes you feel lazy and it makes you run away from things that need to get done.”
Prioritizing intimate relationships	6 (2.8)	“Now I’m married and have a 1 year old child and my wife hates me vaping especially since she found out how poisonous the vape juice is for children and she’s always concerned about me accidently leaving my vape around our child.”

#### Barriers to Quitting

Barriers to quitting were reflected in 35.91% (441/1228) of the posts. Nine barriers were found, which were listed in the order of most saturated to least saturated (Table 3). The most challenging barrier was related to nicotine withdrawal, with reports of intense withdrawal symptoms associated with trying to quit. The second most common barrier was related to nicotine dependence, with many users citing heavy addiction because of the nicotine salt devices that many of the posters were using (eg, JUUL). The third barrier was related to proximity to others who vape as a major barrier to quitting, which included both web-based (eg, seeing friends vaping on social media) and offline interactions. The fourth barrier to quitting was related to existing mental health challenges, whereby users described how their stress, anxiety, or depression made it harder for them to quit. The fifth barrier was related to the use of other substances, including smoking traditional cigarettes and other drugs. The sixth barrier was related to vaping becoming habitual and associated with routine things such as commuting to school or work. The seventh cited barrier was related to the accessibility of vaping from both a purchase standpoint as well as from a use standpoint, as users could vape in areas where smoking is prohibited. Other barriers to quitting included the enjoyment of vaping and experiencing weight-related issues after stopping.

**Table 3:** Barriers to quitting (N=441).

Barrier		Values, n (%)	Representative quote
**Intense withdrawal symptoms**			
	Insomnia; fatigue; sweats; headache; brain fog; chest pain; cough; sore throat; dizziness; anxiety; depression; irritability	137 (31.1)	“I’m 2 days in from daily use but my pulse is around 90-100 bpm when I’m just sitting is this normal? People already told me being anxious 24/7 is common but I feel like my heart won’t stop beating fast all day and night. I feel like I can’t sleep because I’m so anxious about nothing and my heart won’t stop beating quick.”
**Dependency on high nicotine**			
	JUUL; using >20mg/ml	116 (26.3)	“I’m telling you, the nic salts will get you every time. Juuls are just icing on the cake.”
**Proximity to those who vape**			
	Face-to-face interactions; web-based interactions	41 (9.3)	“I used to see vapes on peoples social media and I would get insane cravings out of nowhere.”
**Mental health challenges**			
	High stress; suffer from anxiety or depression	39 (8.8)	“Convinced myself it would help with the stress and anxiety of everything going on.”
**Other substance use**			
	Smoking cigarettes; other drugs	34 (7.7)	“Add in that I took Adderall daily, which if anyone doesn't know, makes your desire to smoke increase x10000000. So breaking into pod #3 in a day wasn't unheard of.”
**Habit or routine associations**			
	Commute; other day-to-day habits	25 (5.7)	“I’m a delivery driver and I think I connect vaping with driving also. But of course now, I vape all the time. In bed, at least 20 minutes after waking up, want it after a meal and an absolute must have when I’m drinking alcohol.”
**Accessibility of e-cigarettes**			
	Easy to purchase; can use anywhere	19 (4.3)	“Vaping I did 10x more than smoking! So easy to do anywhere.”
**Other**			
	Enjoyment of vaping	13 (2.9)	“Also a non-drinker and I feel it’s my only guilty pleasure [...] I haven’t tried to stop yet.”
	Increased appetite or weight gain after stopping	13 (2.9)	“When does the binge eating stop? I've been 2 weeks nicotine free and still wanna eat constantly and gained so much weight.”

#### Facilitators to Quitting

Approximately 46.42% (570/1228) of posts provided a description of facilitators to quit vaping (Table 4). Nine facilitators to quitting were identified. The most cited facilitator to quitting was distractions, with the number one distraction strategy relating to keeping their minds and hands busy through hobbies, movies, games, and mindfulness exercises. Another common distraction strategy was by replacing puffing on an e-cigarette with eating, chewing gum, drinking water or tea, or chewing on flavored toothpicks. Finally, users spoke about the key role that exercise plays in their quitting efforts from running to weight lifting to yoga.

The second most cited facilitator to quitting was related to having a positive mindset or a positive view of self. Individuals described the need to believe in themselves, encourage themselves, care for their mind and body, grace with themselves, and reward themselves. In addition to self-love was the value of social support for quitting vaping. Although the primary source of support drawn upon was others in the Reddit community, some described the positive support received from family, friends, and even quit buddies to help them quit.

Making vaping less available or desirable was another facilitator to quitting vaping, which was achieved through a variety of strategies, including tossing the device, having someone hide the device, and purposely using undesirable flavors to dissuade them from vaping. Another facilitator to quitting was through the use of behavioral support interventions, which was primarily sought through the *Quit Vaping* app, followed by Alan Carr’s book “Quit smoking the easy way,” and then websites that provide information on vaping cessation.

Experiencing the negative effects of vaping, being reminded of their reasons to quit vaping, experiencing the benefits of quitting, and changing their environment (eg, staying away from people who vape, switching up routine, and taking a vacation) were also listed as facilitators to remain vape free.

**Table 4:** Facilitators to quitting (N=570).

Facilitator			Values, n (%)		Representative quote
**Distractions**					
	Keeping mind or hands busy (hobby, entertainment like movies and gaming, and mindfulness exercises); eating, chewing gum, drinking water or tea, and toothpicks; exercise	279 (48.9)		“I am trying to just live in the moment instead of having to pacify every single discomfort with nicotine. A workout program I follow always says ‘get used to the discomfort’ and that thought has been on repeat in my mind. Deep breaths and meditation have been helping a lot too.”	
**Positive mindset or positive self-concept**					
	Resilient mindset; progress mindset (winning streak); self-care, grace, or rewarding	63 (11.1)		“This time feels a lot different though. I am being nice to myself. Letting myself be irritated, eat as many snacks as I want, and really feel my feelings.”	
**Social support**					
	Support from others (Reddit community, friends, and family); quitting with someone	54 (9.5)		“This little community has made a huge difference on my mindset on quitting!! We all want the best for each other.”	
**Making vaping less accessible or less desirable**					
	Tossing device; having someone hide device; using undesirable flavors	54 (9.5)		“When I used zero [nicotine], I just kept my device somewhere completely across the house so it was never instantly accessible. It’s much easier to say no when you think about how little you actually get for the effort of going to grab it.”	
**Behavioral support**					
	App (*Quit Vaping*); Allen Carr *Quit smoking the easy way;* quit help websites	34 (6.0)		“And use some app like quit vaping [app name]. Being able to easily see milestones and achievements is very motivating at the start.”	
**Other**					
	Awareness of negative effects of vaping	23 (4.0)		“I always thought it helped with my anxiety but it honestly only ever made it worse.”	
	Reminders of reasons to quit	17 (3.0)		“I was actually in one of those zones thinking, ‘It wasn’t so bad, maybe I just “like” vaping’ when I started writing this post. By the end of writing it, I remembered how bad my experience really was and haven't had a craving since.”	
	Experiencing benefits of not vaping	11 (1.9)		“I quit two months ago and have only recently started to really feel the positive benefits, but from what I've experienced, it's totally worth it.”	
	Changing environment (social or routine)	11 (1.9)		“I am moving into a house with some very supportive people and I’ve found that when in a new setting, cravings are a lot lower.”	

## Discussion

### Principal Findings

This study is the first of its kind to examine a quit vaping community on Reddit to understand the process of quitting vaping from the perspective of e-cigarette users. The findings of this study shed light on what quitting vaping is like and how it aligns with quitting smoking. The findings reveal that the process of quitting vaping with nicotine, although similar to quitting combustible cigarette smoking in many respects, is also unique in several noteworthy ways.

### Quitting Methods

Both cold turkey and gradual reduction are relevant to vaping cessation. However, significantly more e-cigarette users opted for gradual reduction compared with cold turkey. This is unique compared with smoking, whereby most smokers opt for the cold turkey approach [21]. Interestingly, a large portion of those who chose gradual reduction chose to do so by tapering their vaping device. The fact that e-cigarette users are trying to figure out how to quit on their own in this way reflects the dearth of evidence-based support available to those who want to quit and is indicative of the urgent need to develop resources and guidelines to help e-cigarette users with quitting. There are concerns about e-cigarette users turning to their devices to quit. One particular concern is that of ongoing use. In studies that examined the efficacy of using e-cigarettes to quit smoking, although some users were able to successfully quit smoking, almost all were still vaping a year later [22,23], indicating that the use of e-cigarettes promoted ongoing addiction to nicotine compared with the use of approved pharmacological cessation support (eg, NRT).

Similar to this study, an analysis of >3000 Twitter tweets revealed that a gradual approach to quitting by JUUL users was popular, especially by tapering the nicotine concentration of their pods or changing their devices [24]. In contrast, adolescents in the study by Kong et al [25] used the cold turkey approach more frequently. This difference may be because of the length of nicotine product use, where adolescents may more easily quit cold turkeys because of a shorter timeline of use. The differences found indicate that guidelines and interventions should be tailored to different age groups, with attention to the time using nicotine products as well as the type of products used. The use of high-nicotine salt devices such as JUUL appears to lead to more intense addiction, which may make a cold turkey approach more difficult. The variability of nicotine concentration in vaping devices leads to complexity in relation to cessation, which is different from combustible smoking.

### Reasons for Quitting

In this study, the most commonly cited reasons for quitting vaping were related to experiences of negative health consequences and addiction. Several studies reveal that experiencing negative health consequences and dependence are consistently among the top few reasons for quitting e-cigarettes [3,5,24,25]. Of particular note is the finding around current health effects. In an analysis of 2000 text responses to a question about reasons to quit vaping from the evidence-based e-cigarette cessation program for youth and young adults, “This is Quitting,” researchers found similar findings [3]. They found that the top-rated reason for quitting was health, especially current and general health, compared with future health [3]. The study by Ungar et al [24] also found that experiencing negative health consequences was the top-rated reason to quit. This reason to quit vaping, which is experiencing negative health consequences in the present, is different from the top-rated reason to quit combustible smoking, which is future health [26]. The fact that e-cigarette users are experiencing adverse health effects early in their vaping trajectory brings forward the need to pay more attention to the immediate health effects of vaping. There needs to be particular attention on the effect of vaping on the lungs given that users in this study primarily reported adverse pulmonary outcomes from vaping, including tight chest, sleep apnea, cough, wheezing, and breathlessness.

### Barriers and Facilitators to Quitting

In this study, users reported intense withdrawal symptoms and dependency on high nicotine levels as the top barriers to quitting. This is not surprising given that the most popular e-cigarette devices enable the delivery of very high concentrations of nicotine to the brain (eg, JUUL is typically approximately 50 mg/ml) [27], the amount of nicotine that you would expect to see a heavy smoker consuming. These high-nicotine devices appear to be particularly problematic when trying to quit. This is not surprising given that the largest population of e-cigarette users are youth and young adults [28,29], those at an age when the brain is still developing [30,31], and that a developing brain is most vulnerable to long-term and intense nicotine addiction, which makes quitting much harder [27,31-33]. Countries such as Canada, which are taking a strong stance in limiting the nicotine concentration of e-cigarettes [34], are playing a critical role in protecting developing brains from early and long-term addiction. The findings of this study confirm that taking action to limit the nicotine concentration of these devices is critical. However, cessation services and advice must be reflective of the current state of use, which is when high-nicotine concentrations are allowed, as well as be ready to adapt to future use. Researchers should explore and evaluate how cessation advice and support may best accommodate shifts in use behaviors because of policy changes.

It is also interesting that not only offline interactions but also web-based interactions with other e-cigarette users served as a key barrier to quitting. Vaping has progressed in popularity at a time when social media use and social media influencers are rampant. e-Cigarette companies have successfully tapped into this trend and are not only lending to the uptake of e-cigarettes [35] but are also serving as a barrier to quitting, as revealed in this study. Vaping cessation efforts also need to capitalize on these web-based networks, and there are some innovative efforts being made in this regard (eg, This is Quitting by the Truth campaign) [7,36].

The findings also reveal that mental health challenges are a major barrier to quitting. Tobacco use is known to coincide with mental health issues, including among young adults [37]. Mental health disorders are increasing and are an urgent concern [38], especially in the context of the COVID-19 pandemic [39]. In light of this, attention to and screening for mental health among e-cigarette users is warranted. In addition, cessation interventions that promote positive mental health appear to align with stated facilitators to quitting, which include resiliency, self-grace, and self-love.

Another barrier to quitting is related to the accessibility of e-cigarettes. Vaping with nicotine is easily accessible via web-based sales [40], as well as the recent establishment of a large number of vape retailers that sell these products [41]. For example, in Canada, it was found that 76% of retail outlets sold vaping products in 2014 [42]. In the province of British Columbia alone, there are an estimated 90,000 vape retailers compared with only 6000 retailers that sell combustible cigarettes [43]. In addition, the policies around vaping are different from smoking; for example, you cannot be smoking indoors, but you can still vape in many indoor spaces despite several jurisdictions implementing vape-free indoor policies [44]. This is combined with the discreet and appealing designs of e-cigarettes and that e-cigarette users are left to their own devices to regulate their use. e-Cigarette advertising capitalizes on these freedoms associated with vaping and promotes users to use their vape at all times [45]. For example, a Blu commercial spends the entire 60 second advertising time to tell potential e-cigarette users that they can “smoke blue virtually anywhere” [46]. According to the users who posted on the Reddit community, being able to vape anywhere at any point in time has left them doing exactly that. This constant use has left them feeling “stuck to the vape” and is the primary reason for quitting. These findings have important implications for banning advertisements that promote vaping in this way, as well as policies for limiting where vaping can occur. The findings also hold implications in limiting the accessibility of both web-based and offline sales of e-cigarettes.

The top facilitator to quitting was the use of distractions, which ranged from things that kept the mind and hands busy to replacing the e-cigarette with other things, such as toothpicks and exercise. Similar to smoking cessation interventions such as Crush the Crave [47], the importance of including tips and opportunities to distract those who are trying to quit vaping is key. There is an opportunity for cessation interventions to capitalize on new digital technologies available to develop distractions for young vapers. Given that youth and young adults, the highest demographic of vapers, are ubiquitous on the web, focusing on innovative efforts in this context to help distract them through their cravings would likely prove quite fruitful, especially given their receptivity to receiving interventions for cessation through these media [5].

### Future Research

These findings bring forward several areas for future research. First, the unique aspects of vaping cessation call for urgent research focused on developing cessation guidelines tailored to e-cigarette use. The development of these guidelines should establish both pharmacotherapy and behavioral support recommendations. For pharmacotherapy, it is interesting that a large portion of vapers used NRT to support their quit attempts, and they primarily used the nicotine patch and gum in tandem. There is a need to explore the right *recipe* for vapers who are trying to quit. This is especially critical, as the common use of high nicotine concentrations in e-cigarettes may mean that vapers need a different approach to NRT compared with smokers. For behavioral support interventions, the reported reasons for quitting, barriers and facilitators to quitting, and benefits of quitting could inform the development of interventions that resonate with vapers. Indeed, the findings offer a beginning framework and structure for exploring and developing recommendations.

Longitudinal research that tracks how cessation is approached is needed. It would be interesting to explore whether approaches to quitting shift over time with the introduction of new resources and policies. For example, whether fewer vapers resort to tapering their own devices to quit if pharmacotherapy options are tailored to vaping. It would also be interesting to conduct research on which facilitators are linked to successful long-term abstinence rates and for whom so that various populations have access to more tailored options to help them quit. Finally, there is a need to explore predictors of vaping so that we can identify high-risk populations before engaging in e-cigarette use.

### Limitations

The findings of the study are based on data during a time when there was a pandemic, which may have influenced the experience of quitting. In addition, this study was conducted when few resources were available to support the cessation efforts of e-cigarette users. In addition, the findings are limited to data provided on one platform during a 4-week period. Another limitation of this study is the inability to definitively determine the demographic details of the Reddit community users, limiting the ability to note nuances in experiences and preferences based on demographic variables. However, Reddit users are known to skew toward being young and male [48], indicating that the results may represent the experiences of young males more than any other group. Finally, we could not determine how many users were still vaping and how many were vape free nor we could link particular facilitators to quitting success.

### Conclusions

The findings of this study reveal the unique aspects that encompass the process of quitting vaping from the perspective of e-cigarette users. This work not only validates the need for more stringent policies around e-cigarette consumption and use but also brings forward some important gaps in relation to efforts to meet the needs of e-cigarette users who want to quit. The findings offer some recommendations and a beginning framework for steering e-cigarette policy and intervention efforts so that they resonate with e-cigarette users.

## Abbreviations

NRT: nicotine replacement therapy
